# Efficacy of ultrasound guided venous cannulation positioning during venous-arterial extracorporeal membrane oxygenation

**DOI:** 10.3389/fcvm.2025.1656101

**Published:** 2025-10-16

**Authors:** Yuanyuan Sun, Chengmin Huang, Weimei Ou, Zhixian Liu, Guangfeng Sun, Xinchen Zhang, Xu Chen, Bin Wang, Guoming Zhang

**Affiliations:** ^1^ Department of Ultrasound, Xiamen Cardiovascular Hospital of Xiamen University, School of Medicine, Xiamen University, Xiamen, China; ^2^ Department of Emergency, Xiamen Cardiovascular Hospital of Xiamen University, School of Medicine, Xiamen University, Xiamen, China

**Keywords:** ECMO, ultrasound guidance, venous cannulation, hemodynamics, infection

## Abstract

**Objective:**

To evaluate the clinical impact of ultrasound-guided venous cannulation positioning during the initiation of venous-arterial extracorporeal membrane oxygenation (VA-ECMO).

**Methods:**

This retrospective study included 48 patients who received bedside VA-ECMO support between June 2019 and August 2024. Patients were divided into an ultrasound-guided group (UG, *n* = 23) and conventional body surface landmark group (BSL, *n* = 25). Clinical outcomes, cannula positioning accuracy, complications, infection markers, and prognosis were compared. A subgroup analysis was performed in patients who did not undergo cardiopulmonary resuscitation (non-CPR).

**Results:**

Compared to BSL group, patients in the UG group had significantly higher rates of optimal venous cannula positioning (*p* < 0.01), lower incidence of unstable flow and pulmonary edema, and shorter aortic valve closure time, infection markers (WBC, PCT) were also significantly lower in the UG group (*p* < 0.05). In the non-CPR subgroup, the UG group had shorter ECMO duration, hospital stay, and dual antibiotic therapy duration (all *p* < 0.05), with non-significant trends toward better survival.

**Conclusion:**

Ultrasound-guided venous cannulation improves cannula positioning accuracy, reduces early complications, and may enhance clinical outcomes, particularly in non-CPR patients. Routine use of ultrasound guidance is thus recommended in bedside VA-ECMO procedures.

## Introduction

Extracorporeal membrane oxygenation (ECMO) has emerged as a pivotal life-support modality for patients experiencing severe cardiac and/or respiratory failure (1–3) Since its inception in the 1960s, ECMO has evolved from a perioperative adjunct in cardiac surgery to a widely adopted rescue therapy in various critical care scenarios, including acute respiratory distress syndrome (ARDS), cardiogenic shock (CS), and cardiac arrest (4–8). Its utility was particularly underscored during the COVID-19 pandemic, where ECMO served as a vital intervention in managing patients with refractory respiratory failure (9), and veno-venous ECMO (VV-ECMO) saw the biggest expansion, and the VA also, both modalities being directed to their respective indications. With ongoing advancements in medical technology and rising clinical demand, the application of ECMO, especially venous-arterial ECMO (VA-ECMO), which offers both cardiac and respiratory support, continues to expand globally (10–13).

**Figure 1:**
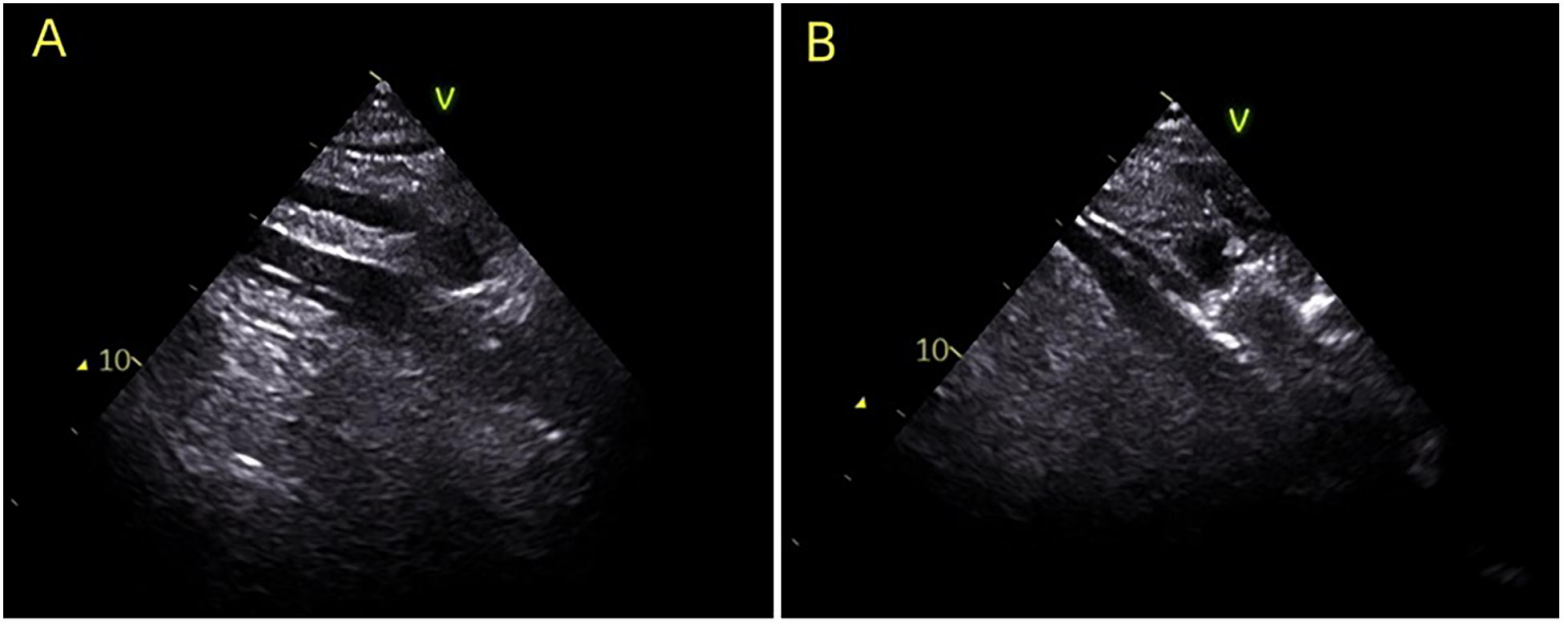
During the establishment of ECMO, ultrasound guidance was utilized to ensure accurate placement of the venous cannula in the ideal position. **(A)** Indicates the guide wire positioned in the right atrium; **(B)** illustrates the venous cannula guided into the right atrium.

Establishing effective VA-ECMO support hinges on the accurate placement of cannulas, particularly the venous cannula, which is essential for maintaining adequate drainage and ensuring stable extracorporeal flow. Misplacement can lead to serious consequences: cannulas positioned too shallowly within the inferior vena cava may adhere to vessel walls and compromise drainage, while those inserted too deeply into the right atrium or superior vena cava may cause atrial or vascular trauma, potentially resulting in catastrophic hemorrhage (5, 14–16). The optimal position for venous cannulation is in the mid-right atrium, balancing efficient drainage with reduced procedural risk. While digital subtraction angiography (DSA) offers ideal visualization for cannula positioning, its availability is often limited, particularly during emergent bedside procedures. When time permits, cannulation is performed under DSA guidance; if time does not allow, bedside implantation is performed. Ultrasound guidance has been increasingly adopted in critical care procedures for its real-time visualization and bedside applicability.

At the bedside, arterial and venous punctures are almost always performed under ultrasound guidance. However, not all patients had their venous cannula tip advanced into the mid–right atrium under ultrasound guidance. However, despite its theoretical advantages, evidence supporting its routine use for guiding venous cannulation into the middle of the right atrium during VA-ECMO initiation remains sparse, and comparative studies are lacking. In order to clarify the clinical impact of this latter step, we conducted the present study.

In this study, we retrospectively analyzed VA-ECMO cases performed at our institution to evaluate the clinical utility of ultrasound-guided venous cannulation. We aimed to determine whether ultrasound guidance improves cannulation accuracy, enhances flow stability, and reduces complications compared to traditional anatomical landmark-based methods.

## Methods

### Study design and population

We conducted a retrospective analysis of 48 patients (36 males, 12 females; age range 32–78 years; mean age 57.52 ± 14.31 years) who underwent bedside VA-ECMO support in the Emergency Department of Xiamen Cardiovascular Hospital, Xiamen University, between June 2019 and August 2024. Inclusion criteria were: (1) clinical diagnosis of cardiogenic shock, electrical storm, or cardiac arrest; and (2) initiation of VA-ECMO support via peripheral femoral cannulation at the bedside. Exclusion criteria were: (1) cannulation performed under digital subtraction angiography (DSA) guidance in a catheterization laboratory; or (2) early termination of ECMO for non-medical reasons. The protocol was approved by the Institutional Ethics Committee (2025-4).

### Grouping and cannulation procedure

Patients were categorized into two groups based on the method of venous cannulation positioning: an ultrasound-guided group (UG, *n* = 23) and a body surface landmark group (BSL, *n* = 25). As shown in Figure 1, in the UG group, real-time ultrasound guidance using a phased-array cardiac probe was employed to track guidewire advancement into the right atrium, and the cannula was subsequently adjusted to ensure placement in the mid-right atrium. In contrast, the BSL group underwent cannulation based solely on anatomical surface landmarks, with guidewire insertion and cannula depth estimated by external measurements, without the aid of imaging guidance.

### Subgroup analysis

To further evaluate the effect of ultrasound guidance on infectious complications and prognosis, a subgroup analysis was performed among patients who did not undergo cardiopulmonary resuscitation (non-CPR subgroup). These patients were further stratified into ultrasound-guided (UG-NCPR) and body surface landmark (BSL-NCPR) subgroups. Comparative analyses were conducted to assess differences in infection-related biomarkers and outcome indicators, aiming to elucidate the potential impact of ultrasound-guided cannulation in the non-CPR patients.

### ECMO cannulation and support protocol

All patients received ECMO support using the Maquet (Germany) peripheral cannulation system. Cannulation was performed via the femoral artery and femoral vein. We routinely used ultrasound to verify guidewire position in both the femoral artery and vein before cannulation. The arterial cannula was advanced into the mid-abdominal aorta, and the venous cannula was positioned either at the mid-right atrium (UG group) or at an empirically determined depth (BSL group). The initial ECMO flow rate was set at 50 mL/kg/min. Systemic anticoagulation was maintained with heparin, adjusted according to activated clotting time (ACT) monitoring. All patients receiving mechanical ventilation were managed using synchronized intermittent mandatory ventilation (SIMV). In patients requiring intra-aortic balloon pump (IABP) support, counter-pulsation was maintained at a 1:1 ratio.

### Clinical data collection

Baseline clinical data were collected for all patients, including demographic characteristics, medical history, primary diagnosis, time to ECMO initiation, pre-ECMO arterial blood gas values, electrocardiographic findings, and transthoracic echocardiographic measurements. Post-ECMO parameters recorded within the first 24 h included fluid infusion volume, ECMO flow stability, presence of pulmonary edema on chest radiographs, central venous pressure, follow-up arterial blood gas and echocardiographic data, duration of ECMO support, use of additional mechanical circulatory devices (e.g., intra-aortic balloon pump), laboratory biochemical indices, and occurrence of ECMO-related complications.

### Operational definitions

• Optimal venous cannula position: Defined radiographically as the cannula tip located in the middle third of the right atrium following the establishment of circulatory support.
• ECMO flow stability: refers to stable ECMO machine operation without cannula vibration or marked fluctuations in flow.
• Severe pulmonary edema: Diagnosed when bilateral chest radiographs showed pulmonary infiltrates occupying more than one-third of both lung fields.
• Successful weaning: Defined as hemodynamic stability maintained for at least 48 h after ECMO discontinuation, without the need for newly initiated mechanical circulatory support (continued use of pre-existing IABP was not classified as new support).

### Statistical analysis

Statistical analyses were performed using SPSS software version 22.0 (IBM Corp., Armonk, NY, USA). Continuous variables were expressed as mean ± standard deviation (SD) and compared between groups using independent samples t-tests. Categorical variables were presented as percentages and analyzed using Fisher's exact tests. A two-tailed *p*-value < 0.05 was considered statistically significant.

## Results

### Baseline characteristics

As shown in Table 1, there were no statistically significant differences between the ultrasound-guided (UG) group and the body surface landmark (BSL) group with respect to age, sex, ECMO time, primary diagnosis, timing of mechanical ventilation, or other baseline clinical parameters (all *p* > 0.05), indicating that the clinical basic conditions of the two groups were comparable.

**Table 1:** Comparison of baseline data between the two groups.

Characteristics	UG (*n* = 23)	BSL (*n* = 25)	*χ*^2^/*T* value	*p* value
Gender			2.254	0.133
Male (%)	15 (65.22%)	21 (84.00%)		
Female (%)	8 (34.78%)	4 (16.00%)		
Age(years old)	62.26 ± 15.34	53.16 ± 17.79	1.890	0.065
ECMO time			1.268	0.279
Normal working hours	12 (52.17%)	14 (56.00%)		
Off working hours	11 (47.83%)	11 (44.00%)		
Primary disease				
AMI (%)	15 (65.22%)	14 (56.00%)	0.483	0.566
Myocarditis (%)	6 (26.09%)	3 (12.00%)	0.832	0.279
Aortic valve stenosis (%)	0 (0.00%)	1 (4.00%)	1.106	0.999
Others (%)	2 (8.70%)	7 (28.00%)	0.973	0.140
The condition of patient before ECMO				
ECPR (%)	9 (39.13%)	15 (60.00%)	0.877	0.248
Hypotensive shock (%)	13 (56.52%)	9 (36.00%)	0.912	0.246
Electric storm (%)	1 (4.35%)	1 (4.00%)	1.201	0.999
Lactic acid value before ECMO (mmol/L)	13.00 ± 6.62	11.69 ± 5.31	0.785	0.452
pH value before ECMO	7.11 ± 0.35	7.11 ± 0.32	−0.091	0.928
The duration for establishing ECMO (min)	30.43 ± 10.87	35.75 ± 11.17	−1.671	0.102
The inner diameter of the venous cannula (F)	20.13 ± 1.01	19.64 ± 0.95	1.728	0.091
The inner diameter of the arterial cannula (F)	16.13 ± 1.01	16.04 ± 1.17	0.285	0.777

UG, the ultrasound-guided group, BSL, the body surface landmark group; AMI, acute myocardial infarction; ECPR, extracorporeal cardiopulmonary resuscitation.

### Clinical parameters during ECMO support

As shown in Table 2, post-ECMO chest radiographs confirmed optimal venous cannula positioning in 21 patients (91.30%) in the UG group, compared to only 8 patients (32.00%) in the BSL group, a statistically significant difference (*p* < 0.05). Furthermore, the UG group demonstrated fewer episodes of flow instability, lower cumulative fluid infusion volumes within 24 h post-ECMO initiation, and a lower incidence of severe pulmonary edema (all *p* < 0.05).

**Table 2:** Comparison of clinical data between the two groups during the support period 24 h post-ECMO.

Measurements	UG (*n* = 23)	BSL (*n* = 25)	χ^2^/*T* value	*p* value
Optimal venous cannula positioning (*n*, %)	21 (91.30%)	8 (32.00%)	20.340	0.001
Unstable flow (*n*, %)	10 (43.48%)	18 (72.00%)	4.009	0.045
Fluid infusion volumes (L)	3.99 ± 1.26	5.01 ± 1.72	−2.338	0.024
Severe pulmonary edema (*n*, %)	8 (34.78%)	19 (76.00%)	8.270	0.004
LVEF (%)	21.00 ± 14.58	17.24 ± 10.71	1.024	0.311
Left ventricular diameter (mm)	45.57 ± 9.38	45.48 ± 6.24	0.037	0.970
Aortic closure (*n*, %)	12 (52.17%)	17 (68.00%)	1.255	0.263

UG, the ultrasound-guided group, BSL, the body surface landmark group; LVEF, Left ventricular ejection fraction.

Echocardiographic data collected 24 h after ECMO initiation showed no significant differences between the two groups in terms of left ventricular ejection fraction or end-diastolic dimension (*p* > 0.05). Although the proportion of patients exhibiting aortic valve closure did not differ significantly between groups (*p* > 0.05), the time of aortic valve closure was significantly shorter in the UG group compared to the BSL group (1,833.00 ± 288.70 min vs. 2,372.00 ± 379.08 min, *p* < 0.05), suggesting earlier cardiac unloading.

### Inferior vena cava injury and infection markers

As summarized in Table 3, no cases of inferior vena cava (IVC) injury occurred in the UG group. In contrast, two IVC injuries were reported in the BSL group, both involving cannulation-related vascular trauma and bleeding. Additionally, peak white blood cell (WBC) counts and procalcitonin (PCT) levels were significantly lower in the UG group (*p* < 0.05), suggesting reduced inflammatory response. However, there were no significant differences between groups in the proportion of patients receiving dual antibiotic therapy or in the duration of such therapy (*p* > 0.05).

**Table 3:** Comparison of indicators of inferior vena cava injury and infection between the two groups during hospitalization.

Measurements	UG (*n* = 23)	BSL (*n* = 25)	*χ*^2^/*T* value	*p* value
Injury of the inferior vena cava (*n*, %)	0 (0.00%)	2 (8.00%)	0.920	0.116
The maximum value of WBC (*10^9^/L)	16.17 ± 5.01	25.32 ± 11.35	−3.556	0.001
The maximum value of PCT (ng/mL)	11.10 ± 8.10	25.11 ± 13.49	−4.315	0.001
Cases using dual antibiotics (*n*, %)	17 (73.91%)	23 (92.00%)	2.822	0.093
Duration of using dual antibiotics (day)	6.48 ± 5.98	7.08 ± 7.71	−0.300	0.765

UG, the ultrasound-guided group, BSL, the body surface landmark group; WBC, white blood cell; PCT, procalcitonin.

### Prognostic outcomes

**Figure 2:**
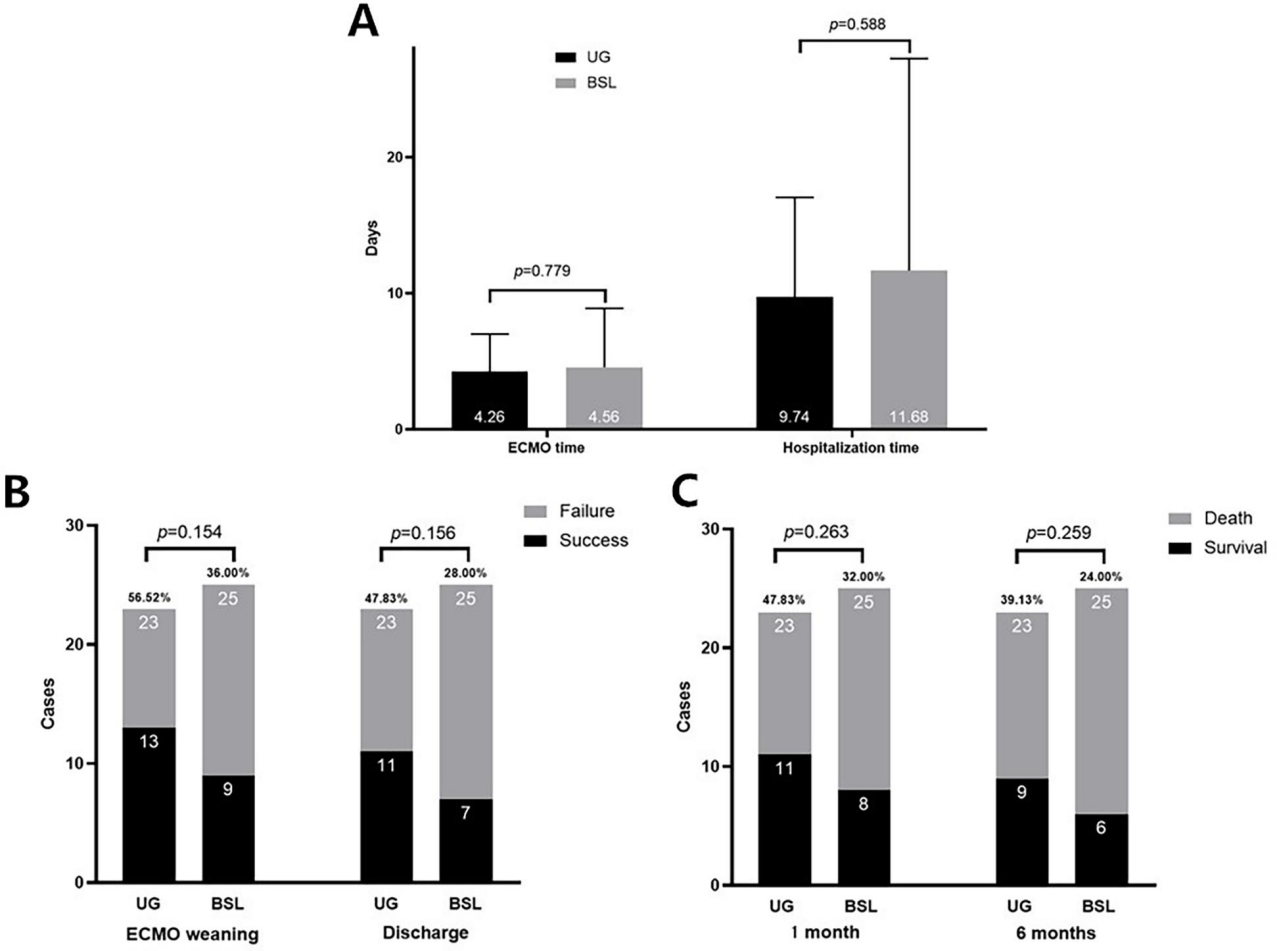
The comparison of clinical outcomes between two groups. **(A)** ECMO Support Duration and Hospital Stay: The duration of ECMO support and length of hospital stay were comparable between the two groups (*p* > 0.05). **(B)** ECMO Weaning Success and Hospital Discharge Rates: The differences between two groups were not significant (*p* > 0.05). **(C)** One-Month and Six-Month Survival Rates: The differences between two groups were not significant (*p* > 0.05).

Figure 2 illustrates the clinical outcomes for both groups. No significant differences were observed between the UG and BSL groups regarding total duration of ECMO support or hospital stay (*p* > 0.05). The differences of rates of successful weaning, hospital discharge, and one- and six-month between two groups survivalwere not significance (*p* > 0.05).

### Subgroup analysis in non-CPR patients

Considering the typically poor prognosis of patients requiring ECMO after cardiopulmonary resuscitation (CPR), a subgroup analysis was conducted among patients who did not undergo CPR (UG-NCPR vs. BSL-NCPR). This cohort included 14 patients in the UG-NCPR group and 10 patients in the BSL-NCPR group. As presented in Table 4, the UG-NCPR group had significantly lower peak WBC counts (*p* = 0.006) and PCT levels (*p* = 0.001), along with a significantly shorter duration of dual antibiotic therapy (*p* = 0.001).

**Table 4:** Comparison of indicators of infection between the two subgroups during hospitalization.

Measurements	UG-NCPR (*n* = 14)	BSL-NCPR (*n* = 10)	*χ*^2^/*T* value	*p* value
The maximum value of WBC (*10^9^/L)	15.71 ± 4.45	29.10 ± 15.69	−3.049	0.006
The maximum value of PCT (ng/mL)	9.37 ± 8.25	31.08 ± 17.12	−4.143	0.001
Cases using dual antibiotics (*n*, %)	9 (64.29%)	8 (80.00%)	0.697	0.404
Time of using dual antibiotics (day)	6.38 ± 3.32	12.50 ± 8.24	−2.535	0.019

UG-NCPR, the ultrasound-guided group of none-CPR patients before ECMO, BSL-NCPR, the body surface landmark group of none-CPR patients before ECMO; WBC, white blood cell; PCT, procalcitonin.

**Figure 3:**
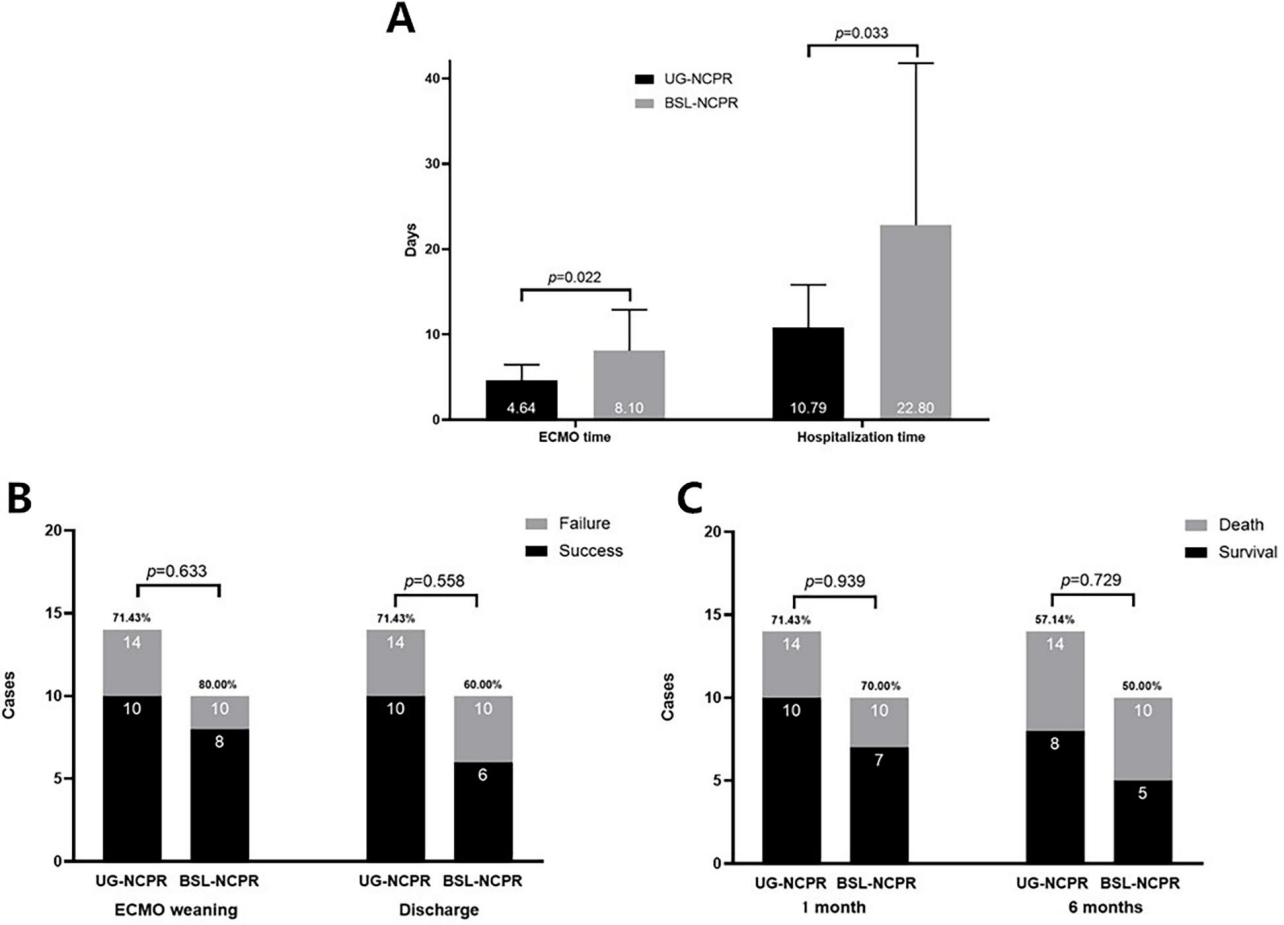
The comparison of clinical outcomes between the two sub-groups. **(A)** ECMO Support Duration and Hospital Stay: The duration of ECMO support and length of hospital stay of the UG-NCPR group were shorter than those of BSL-NCPR group (*p* < 0.05). **(B)** ECMO Weaning Success and Hospital Discharge Rates: The differences between two groups were not significant (*p* > 0.05). **(C)** One-Month and Six-Month Survival Rates: The differences between two groups were not significant (*p* > 0.05).

Moreover, as shown in Figure 3, patients in the UG-NCPR group had shorter durations of ECMO support (*p* = 0.022) and hospital stay (*p* = 0.033) compared to those in the BSL-NCPR group. The difference of rates of successful weaning, discharge, and 1- and 6-month survival between two groups were were not significant (all *p* > 0.05).

## Discussion

ECMO remains a cornerstone in the management of patients with refractory cardiogenic shock. The establishment of stable and adequate extracorporeal flow is critical to its effectiveness. Excessive flow may increase left ventricular afterload, hindering myocardial recovery (8, 17–19), while insufficient flow results in inadequate perfusion and failure to resolve oxygen debt, thereby compromising therapeutic efficacy (2, 20). In addition to flow rate modulation, cannula positioning—particularly the venous cannula—plays a central role in maintaining effective ECMO performance. Cannulae with multiple side holes enhance drainage; however, when positioned too low within the inferior vena cava, suction-related adherence to the vessel wall may occur, reducing venous return. Conversely, placement within the mid-right atrium mitigates this risk and optimizes drainage dynamics.

In clinical practice, especially in urgent bedside settings, cannulation is often performed without fluoroscopic guidance. Traditional reliance on external anatomical landmarks lacks precision, increasing the risk of malposition. Although case reports have described the feasibility of ultrasound-assisted venous cannulation, comparative studies evaluating its efficacy against conventional methods remain limited (21–23). Our retrospective review over a five-year period provides evidence supporting the utility of ultrasound guidance in improving procedural accuracy and reducing complications.

In our study, baseline characteristics were comparable between the ultrasound-guided (UG) and body surface landmark (BSL) groups, confirming the reliability of between-group comparisons. Importantly, the time required for ECMO establishment did not differ significantly, indicating that real-time ultrasound did not delay cannulation. However, significantly more patients in the UG group achieved optimal cannula placement in the mid-right atrium, with a concomitant reduction in early fluid infusion volume. This suggests improved venous drainage and more efficient oxygen debt repayment, potentially minimizing the risk of North-South syndrome. In contrast, inadequate venous return may necessitate compensatory increases in flow, predisposing patients to left heart distension and delayed myocardial recovery.

The UG group also experienced a lower incidence of moderate to severe pulmonary edema within 24 h post-cannulation, further supporting the hemodynamic advantages of accurate venous positioning. Infection-related markers, including peak leukocyte count and procalcitonin levels, were significantly lower in the UG group, suggesting a potential reduction in systemic inflammatory response (24, 25). While the overall cohort did not show significant differences in antibiotic exposure, subgroup analysis of patients who did not undergo cardiopulmonary resuscitation (CPR) revealed a significantly shorter duration of dual antibiotic therapy in the UG group. These findings imply that precise venous cannula positioning may improve pulmonary fluid management, reduce infection risk, and thereby decrease antibiotic burden.

Although no statistically significant differences in ECMO duration, hospital stay, or survival outcomes were observed in the overall cohort, the non-CPR subgroup demonstrated clinical advantages with ultrasound guidance. Specifically, patients in the UG-NCPR group had significantly shorter ECMO support and hospitalization durationsThese results are consistent with prior literature suggesting that ultrasound may enhance procedural safety and clinical outcomes during ECMO initiation.

This study has several limitations. Its retrospective, single-center design inherently introduces selection bias. Precise timing of cannulation procedures and measurement of anatomical parameters, such as inferior vena cava diameter, were not uniformly available, limiting the granularity of procedural comparisons. In this study, we used PCT to preliminarily assess the patients' lung infection status. The use of a single evaluation indicator might lead to biased results. Additionally, the small sample size may have restricted statistical power in detecting differences in long-term outcomes. Given the small sample size, selection bias may indeed account for some of the observed differences. Our intention was to provide preliminary insights that may inform clinical management, and we hope that future studies with larger sample sizes will provide more robust evidence.

## Conclusion

Ultrasound-guided venous cannulation during VA-ECMO initiation enhances cannula positioning accuracy and contributes to more stable hemodynamic support, with a lower incidence of early pulmonary edema and reduced inflammatory markers. Among patients not undergoing CPR, ultrasound guidance was associated with reduced infection burden, shorter antibiotic exposure, and shorter ECMO support and hospitalization durations. These findings support the integration of bedside ultrasound into routine ECMO cannulation protocols, particularly in emergency settings.

## Data Availability

The raw data supporting the conclusions of this article will be made available by the authors, without undue reservation.
